# Open-Coil Retraction Spring

**DOI:** 10.1155/2011/435709

**Published:** 2011-09-29

**Authors:** Pavankumar Janardan Vibhute

**Affiliations:** Department of Orthodontia, Sharad Pawar Dental College, Datta Meghe Institute of Medical Sciences, Deemed University, Room no. 101, Sawangi Meghe, Wardha, Maharashtra 442004, India

## Abstract

Sliding mechanic has become a popular method for space closure with developments in preadjusted edgewise appliance. Furthermore, various space closing auxiliaries have been developed and evaluated extensively for their clinical efficiency. Their effectiveness enhanced with optimum force magnitude and low-load deflection rate (LDR)/force decay. With the advent of NiTi springs in orthodontics, LDRs have been markedly reduced. For use of NiTi, clinician has to depend upon prefabricated closed coil springs. “Open Coil Retraction Spring (OCRS)” is developed utilizing NiTi open-coil spring for orthodontic space closure. This paper describes fabrication and clinical application of OCRS which have number of advantages. It sustains low LDR with optimum force magnitude. Its design is adjustable for desired length and force level. It is fail-safe for both activation and deactivation (i.e., it cannot be over activated, and decompression limit of open coil is also controlled by the operator, resp.). A possibility to offset the OCRS away from mucosa helps to reduce its soft-tissue impingement.

## 1. Introduction

 With the development of preadjusted edgewise appliance (PEA) and low-friction brackets, sliding mechanics has become a popular method for space closure [1–3]. Various retraction force techniques have been developed with sliding mechanics such as elastic module, ligature laceback, elastomeric chain, closed-coil spring, and so forth. Most of them have been extensively evaluated for their efficiency [4–14]. Their efficiency enhanced with optimum force magnitude and low-load deflection rate (LDR)/force decay. LDR/force decay has been markedly reduced with development of NiTi. For the use of NiTi, clinician has to depend upon prefabricated closed-coil springs, which are commercially available in a fixed length and with prefabricated hooks. Preparing and incorporating engaging hook into closed-coil spring from spool is again tricky and a complicated task. With miniscrew anchorage, special attachment hooks are obligatory for preformed closed-coil springs. Clinician has to depend upon different grades (light, medium, heavy) or different length (7 mm to 12 mm) of closed-coil springs for varying the force level at different situations.

 This paper describes a procedure for construction and clinical application of “Open Coil Retraction Spring (OCRS).” It serves an efficient auxiliary for space closure in sliding mechanics with PEA and is constructed using NiTi open-coil spring.

## 2. Fabrication and Clinical Application

OCRS is constructed using NiTi open-coil spring. Length of open coil to be taken for construction depends upon the span between anterior (crimped hook on archwire) and posterior (molar hook or miniscrew head) hooks and the amount of extraction space. Thereby, design and fabrication of OCRS is described by taking into consideration the distance between the anterior and posterior hooks to be 25 mm and premolar extraction space of about 8 mm approximately.

**Figure 1:**
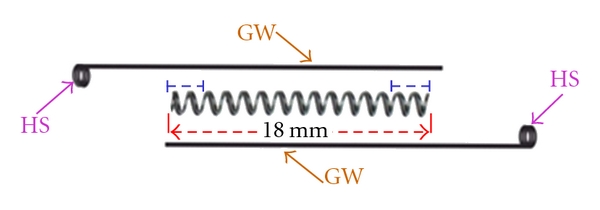
Components in the construction of OCRS: open-coil spring and 0.014′′ SS guide wires (GW) having “helical stop” (HS). Annealed terminal part (marked in blue) of open-coil spring include about two coils.

**Figure 2:**
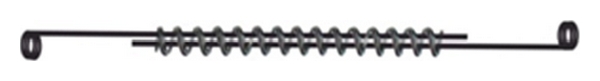
Insert straight parts of both guide wires through both sides of lumen opening of open-coil spring, and pass them through the entire lumen towards each other (in opposite directions).

**Figure 3:**
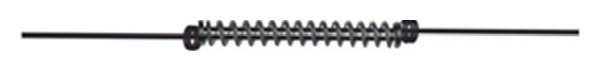
After insertion through the lumen of open coil, straight parts of guide wires also passed inside the “helical stop” of opposing guide wires.

**Figure 4:**
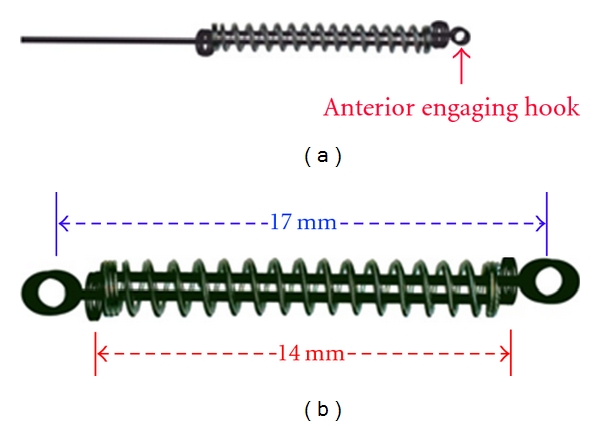
(a) 1st (anterior) engaging round hook prepared in one guide wire. (b) OCRS in its rest position. Although the initial length of the open coil was 18 mm, in final configuration, it is compressed to 14 mm; that is, at rest position of OCRS, open coil is shortened by compression of 4 mm than the original taken length. Total recommended length of OCRS at rest position (distance between two engaging hooks) is about 17 mm.

**Figure 5:**
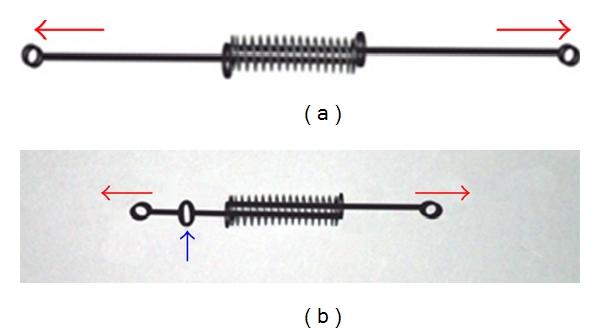
(a) Extent of OCRS activation is checked with stretching both engaging hooks apart, which results in the compression of open coil between two “helical stop”. (b) In one modification of OCRS, “intermediate helical stop” prepared in the middle part of guide wire.

**Figure 6:**
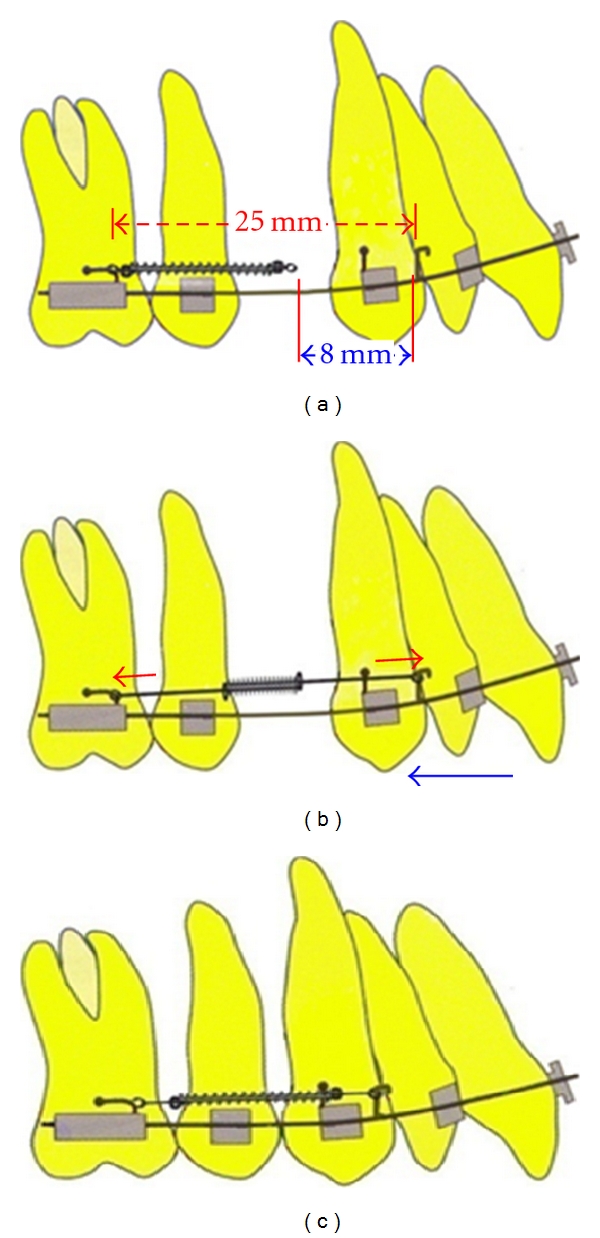
Schematic diagrams depicting modus operandi of OCRS, when applied for the 1st premolar extraction case: (a) at rest position of OCRS, its total length is shorter by 8 mm than total span between molar hook and anterior archwire hook; (b) with activation, open coil gets sandwiched between two “helical stop;” (c) with deactivation of OCRS, space closure is accomplished.

1. Take 18 mm length of NiTi open-coil spring of 0.030′′ lumen size, and anneal its terminal ends (about two-two coils at both ends) (Figure 1).
2. Take two pieces of 0.014′′ stainless steel (SS) wires of about 3.5 cm in length as “guide wires (GW),” and prepare a double helix at one end in each guide wire to form “Helical Stop” (HS). Keep plane of “HS” perpendicular to the long axis of guide wire and its diameter approximately equal to that of NiTi open-coil lumen (Figure 1).
3. Insert both guide wires (straight parts) through open-coil spring from both sides (lumen opening), and pass them through the entire lumen coaxially, towards each other (i.e., in opposite directions) (Figure 2), and also through the “HS” of opposing guide wires (Figure 3). Stretch apart completely penetrated guide wires of both sides; compression of open coil spring in between the two “HS” confirm accurate insertion of guide wires.
4. Prepare 1st round engaging hook in one of the guide wire; doing so, open coil gets compressed from 18 mm to 14 mm. This will be the anterior side of the OCRS for engaging the anterior attachment on arch wire (Figure 4(a)). (Annealing terminal parts of open coil causes the early compression in those parts only, abutting well with “HS”. Thus, poking out of free ends of NiTi open-coil wire is avoided.)
5. Prepare 2nd round engaging hook in another guide wire at the other end of spring. This will be the posterior side of OCRS, for engaging the posterior attachment (molar hook or miniscrew head). Recommended total length between the two hooks of OCRS at rest position is about 17 mm (Figure 4(b)). Although, the initial length of open coil was 18 mm; in final configuration of OCRS, it becomes 14 mm; that is, in rest position of OCRS, open coil is compressed by 4 mm than its original length.
6. Confirm the extent of OCRS activation with stretching both hooks apart (Figure 5(a)).
7. In one modification of OCRS, “intermediate helical stop” may be prepared if controlled and limited amount of space closure or retraction is desired (Figure 5(b)). In cases, where the closing force is not desirable after certain amount of space closure, this intermediate helical stop is helpful as it does not allow further the decompression of open-coil spring.
8. In modus operandi of OCRS, when applied for premolar extraction case, its total length at rest position (17 mm, distance between two round engaging hooks) is about 8 mm shorter than the span between the anterior and posterior points of attachments (crimped hook on archwire anteriorly and molar hook or miniscrew head posteriorly, which is about 25 mm) (Figure 6(a)). During space closure, open coil in OCRS decompresses with approaching towards its rest position (Figures 6(b) and 6(c)).

**Figure 7:**
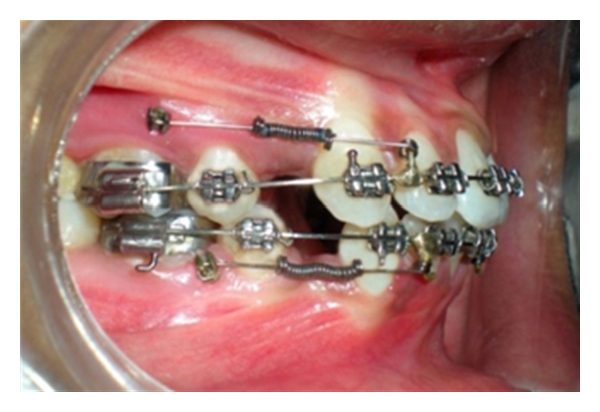
Instead of posterior engaging round hook, posterior guide wire was directly inserted through a ligature hole available in miniscrew head and cinched/bends back.

In case of miniscrew anchorage, prepare posterior engaging round hook adequate in size, so that it engages over the miniscrew head easily. If  ligature hole is existing in miniscrew head, posterior engaging round hook is not necessary, as posterior guide wire can be directly inserted through it and cinched/bend back (Figure 7).

## 3. Case

### 3.1. Diagnosis and Treatment Plan

**Figure 8:**
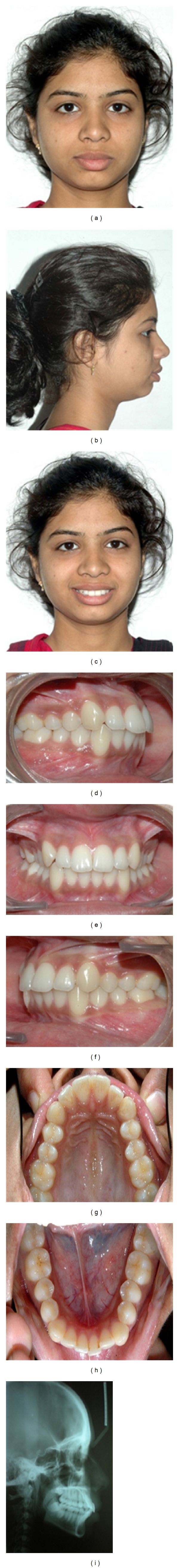
Pretreatment photographic and cephalometric record.

A 20-year-old female presented with chief complaint of bimaxillary protrusion, convex profile, and protrusive lips (Figure 8). She had diagnosed as mild skeletal class II and dental class I malocclusion with bialveolar protrusion and mild horizontal growth pattern. Treatment plan called for 1st premolar extractions to resolve proclination considering as “group B” anchorage case. Use of transpalatal bar was decided for controlling anchorage and OCRS as a space-closing auxiliary.

### 3.2. Treatment Progress

**Figure 9:**
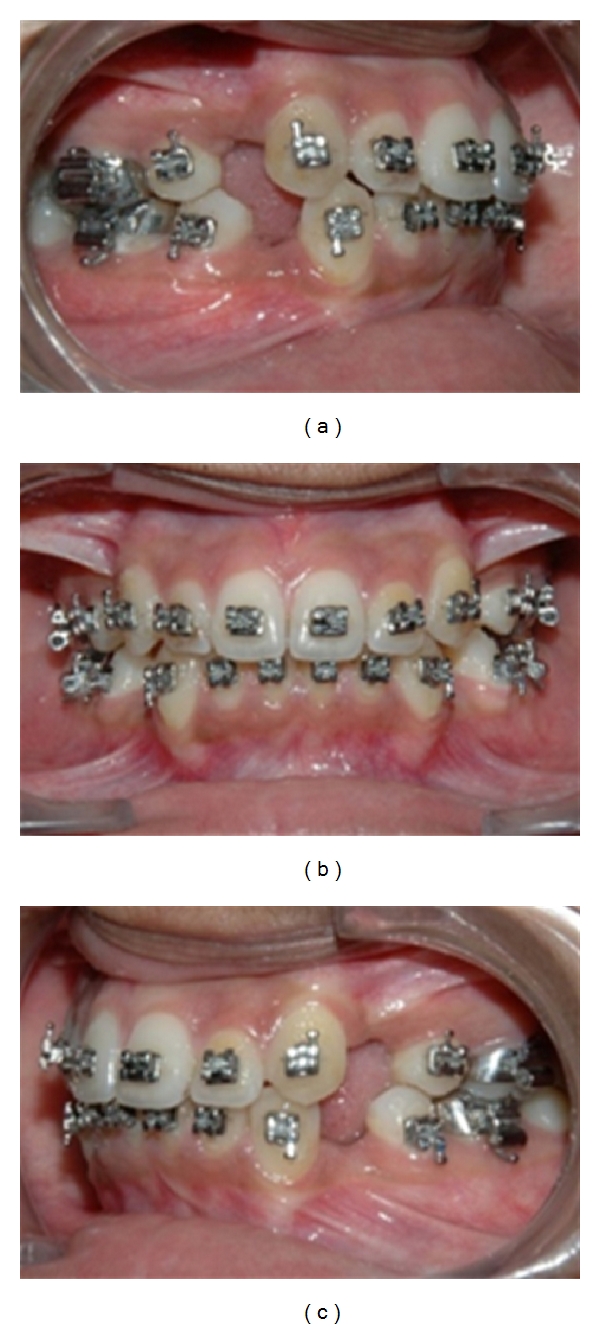
Completion of alignment and leveling.

**Figure 10:**
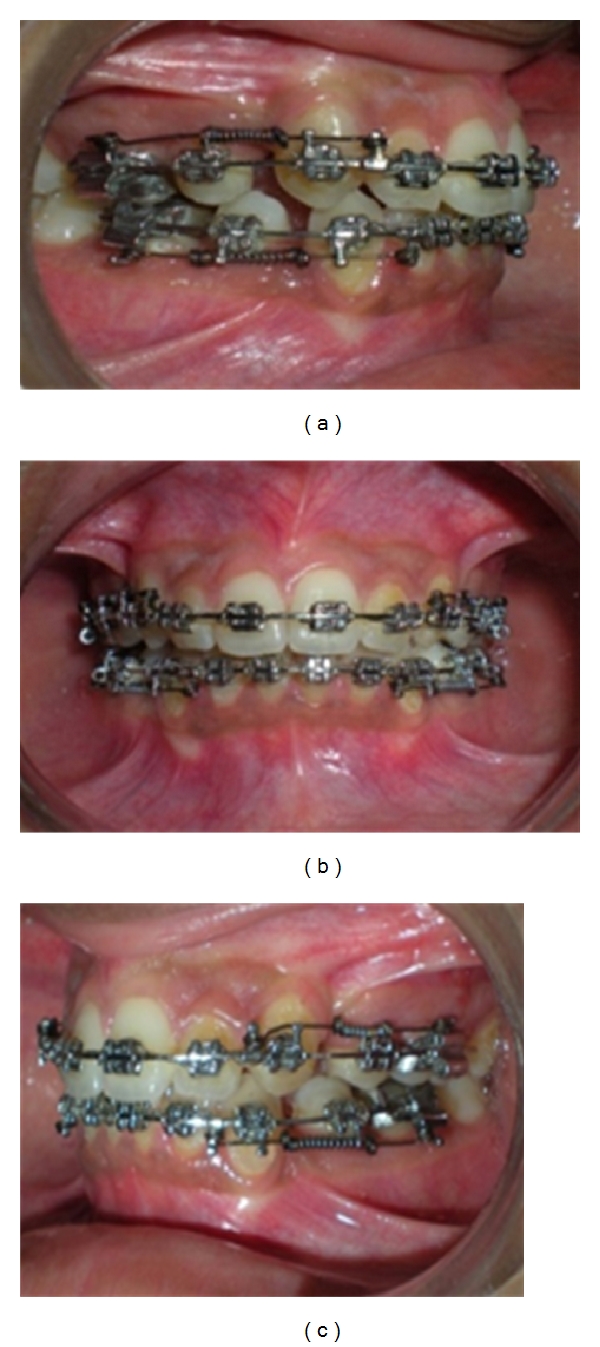
Extraction space closure in progress with OCRS.

**Figure 11:**
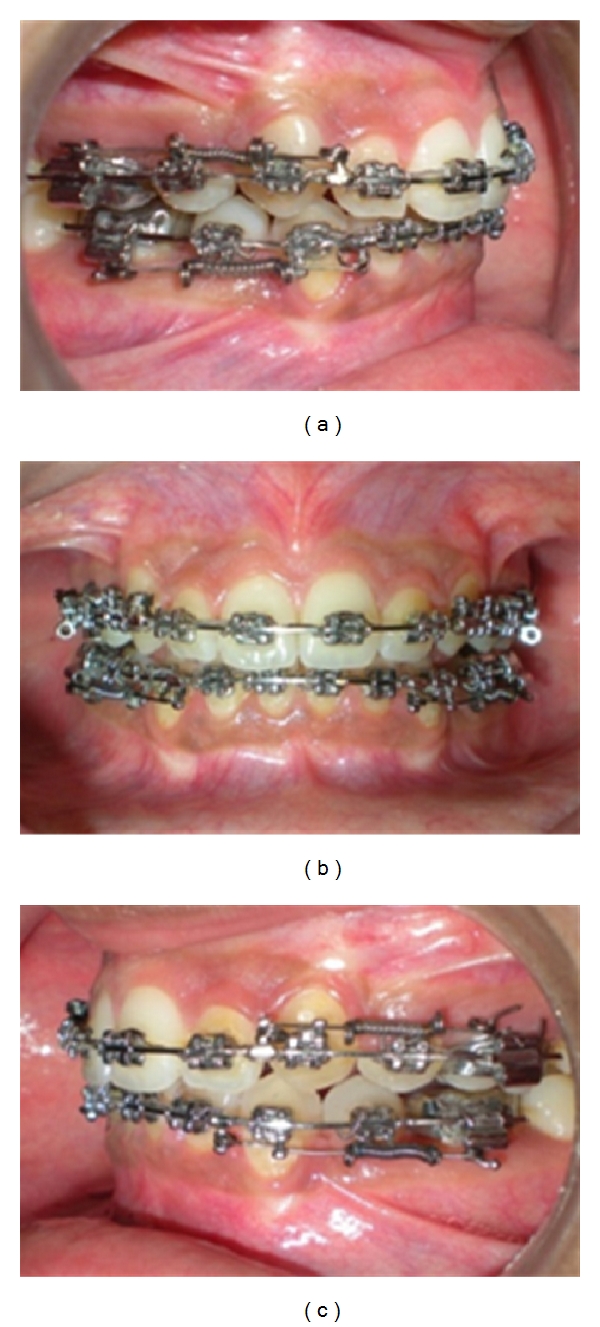
Complete closure of extraction space with OCRS in place.

0.018 PEA brackets were bonded. After two and half months, alignment and leveling were completed in both arches, and four first premolars were extracted (Figure 9). Space closure was started with conventional sliding mechanics and considered as a case of “group B” anchorage. Coordinated arch forms, and precurved 0.016′′ × 0.022′′ SS continuous archwires in both arches were used to prevent the bite from deepening during retraction as per conventional sliding mechanics with PEA. A partly prefabricated custom-made OCRSs were used for en-masse retraction. They were stretched and engaged posteriorly over 1st molar hooks and anteriorly to the hook on archwire (Figure 10). Forces delivered by OCRSs were calibrated and optimized with tension gauge to deliver 250 gm–300 gm. At the end of one year, space closure was completed (Figure 11) without adverse effects, and bimaxillary proclination was resolved. Retraction springs did not show any signs and symptoms of soft tissue irritation and distortion. Upper wraparound and lower Hawley's retainers were delivered.

### 3.3. Treatment Results

**Figure 12:**
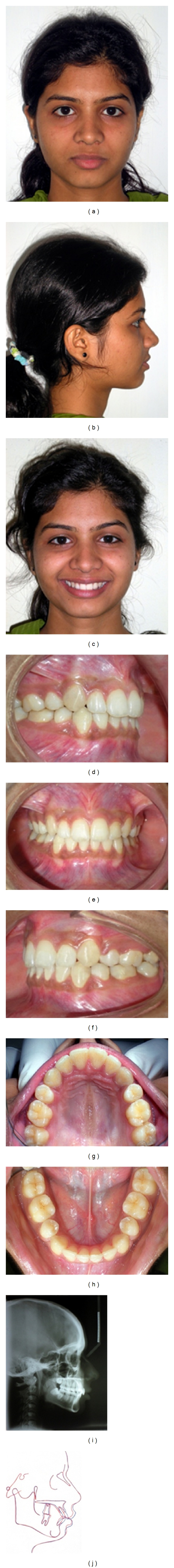
(a–i) Posttreatment photographic and cephalometric record. (j) Pre- and posttreatment cephalometric superimposition.

After 15 months of total active treatment, goals had been achieved. Upper and lower anterior teeth were retracted and uprighted approaching towards their normal position over basal bone, and patient showed good class I dental relationship. Space closure was completed without adverse effects, that is, posterior open bite, deepening of bite. With retraction of upper and lower lips, facial profile was improved (Figure 12). 

## 4. Discussion

 The modus of force generation with the case was decompression of the compressed open-coil spring. This decompression force was used here to generate the definitive traction system. 

Springs with larger lumen size and smaller wire diameter are indicated for orthodontic use because of their more constant force production [15]. Lumen size of open-coil spring (0.030′′) used in OCRS is definitely larger than that of today's commercially available closed-coil springs. Boshart et al. [16] compared the load deflection rates of 10 mm lengths for variety of open- and closed-coil springs made of HiT SS and co-chr. The advent of Japanese NiTi archwires led to the introduction of NiTi coil spring. Miura et al. [17] studied the differences between the Japanese NiTi open- and closed-coil springs and SS springs. The closed-coil springs made of SS showed a linear relationship between load and deflection. The NiTi springs, however, demonstrated a superelastic effect, with a constant load for a large range of deflection. Miura et al. [17] also indicated that open-coil springs deliver a relatively more constant load value in superelastic region than the closed-coil spring. Thus, a more desirable continuous force can be obtained from the open-coil spring than the closed-coil spring. Superelastic activity is evident when the open-coil spring is compressed from 75% to 15%. Miura et al. [17] have shown the clinical applicability of open-coil spring with wire a diameter of 0.012 inch, lumen of 0.030 inch, and 150 gm of superelastic activity when spring was compressed between maxillary central incisor and canine. When the pitch of coils spring is changed from fine to coarse, the load value of superelastic activity can still remain the same, and the range of superelastic activity increases [17]. In the case shown here, OCRS provided the efficient method for the extraction space closure and offered a number of advantages.

**Advantages**

(1) Use of NiTi's open coil maintains low-load deflection rate, while the design is adjustable for desired length and force level. At varied distance between anterior and posterior attachments, the same spring can be adjusted to control the force levels by varying the guide wire length while preparing the 2nd engagement hook. (For excessive span between posterior molar hook and anterior archwire hook, desired magnitude of force may be delivered without over-compression of the OCRS just by increasing the one of guide wire length.) (2) It is fail-safe for both activation and deactivation (i.e., cannot be overactivated and decompression limit of open coil controlled by the operator). (3) A possibility to offset the OCRS away from mucosa reduces its soft-tissue impingement. (4) Size of posterior engaging hook may be customized according to the size and the shape of posterior attachment. In case of miniscrew anchorage, engaging hook is fabricated enough in size so that it engages miniscrew's head easily. Sometime, posterior hook in OCRS may not be required if a hole is present in miniscrew's head. (5) By marking guide wires at specific intervals, force exerted by spring may be calibrated. (6) It is economical than closed-coil spring, and partly prefabrication in stock, reduces chair side time and only require minor adjustments of guide wire length and hook preparation.

With this innovation, clinician does not have to depend only on the conventionally provided approaches, for example, closed-coil springs. OCRS serves as an alternative and take the advantage of low-load deflection rate and long range of action.
